# Action Speaks Louder: The Role of Proactive Behavior between Creative Leadership and Employees’ Creativity

**DOI:** 10.3390/bs14030257

**Published:** 2024-03-21

**Authors:** Xiaozhou Zhou, Jie Zhong, Li Zhang

**Affiliations:** 1 School of Economics and Management, Yanshan University, Qinhuangdao 066000, China; zxzacademic@126.com; 2 School of Management, Harbin Institute of Technology, Harbin 150000, China; 3 School of Economics and Management, Tsinghua University, Beijing 100000, China; zhongjie@sem.tsinghua.edu.cn

**Keywords:** creative leadership, proactive behavior, conformity value, employee creativity

## Abstract

Drawing on social learning theory, this study presents a moderated mediation model to examine the role of proactive behavior and conformity value in the positive relationship between creative leadership and employees’ creativity. Two-wave data are collected from 230 employees and their leaders in an automobile manufacturing enterprise in China, in which employees are encouraged to be creative because they need to implement novel designs and proposals to attract consumers. Statistical analysis reveals that proactive behavior partially mediates the influence of creative leadership on employees’ creativity. Conformity value moderates not only the relationship between creative leadership and proactive behavior but also the indirect effect of creative leadership on employees’ creativity via proactive behavior. The relationship and the mediating mechanism are stronger in the presence of employees’ higher conformity value. We discuss the theoretical and practical implications as well as future research directions of the findings.

## 1. Introduction

Employees’ creativity, which refers to the production of employees’ innovative and useful ideas for products, services, and management processes of a company [1,2], is considered an important determinant of organizations’ survival and growth [3]. Creativity scholars have argued that creativity is a “potent competitive weapon” for organizations [4] and have emphasized that cultivating employee creativity is a major objective for leaders in the 21st century [5]. Previous research has demonstrated that leaders play a key role in cultivating employees’ creativity [6]. Four facets have highlighted the influence of leaders on employee creativity: leaders’ type, leaders’ attributes, leaders’ behaviors, and leader–follower relationships [7,8,9,10].

Little research has focused on how creative leadership influences employee creativity. Despite its potential to promote creativity in organizations, only Koseoglu et al. [7] have explored the positive impact of creative leaders on employee creativity via subordinate creative role identity. However, we have decided to focus on creative leadership rather than creative leaders in our research because it emphasizes leadership style rather than individual leaders themselves. Besides this, an important issue remains unsolved. Existing studies lack attention to the meditating role of employee behavior between leadership and employee creativity. They have mostly investigated the mediating role of psychological mechanisms between leadership and employee creativity [11,12,13,14]. Scholars have called for different theoretical perspectives to explore the mechanism of creativity stimulation [7,13]. Action speaks louder than psychological minds. During an actual implementation process, new ideas continually emerge. In this study, we introduce proactive behavior as the transmission mechanism between creative leadership and employee creativity. Proactive behavior, which is characterized as self-initiated and future-oriented, emphasizing innovation during the implementation process [15,16], can be the driving force in translating creative ideas and potentials into tangible outcomes.

Social learning theory suggests that individuals can learn by observing and imitating the behaviors of their main social cues [17]. Creative leaders constantly search for ways to do things better and promote results [18]. They utilize creativity during working processes, recognize and define problems in novel and useful ways, and solve problems with unconventional methods [19,20]. As representative role models for employees in the workplace [21], employees can observe and learn the attitudes and behaviors of creative leaders and then take proactive steps to improve the status quo, breaking through the constraints of the environment, taking control and bringing positive change to current situations [22].

As for creativity, this refers to employees’ ability to propose valuable and innovative ideas concerning the products, services, and workflows of an organization [2]. Those ideas cannot be generated overnight, but are extracted from long-term practice, because practice is the sole criterion of truth. Proactive behavior is the self-initiated and future-oriented behavior that aims to cause positive change for current situations [15]. When employees have a sincere desire to change the status quo of an organization, they will consider how to make improvements and turn their ideas into practice. During their proactive efforts, employees look for solutions through constant practice that are practical and effective, which also allows their own creativity to develop. In addition, in creativity componential theory, Amabile [23] identified three dominant components of creativity, including domain-relevant skills, creative relevant skills, and intrinsic motivation. The former two are of greater significance, because they become sharpened during problem finding and problem solving [23]. Employees who are keen on proactive behavior tend to gain more practical experience than their passive peers. In the process, they spot, evaluate, and redefine problems; develop ideas; and come up with final solutions [22]. In brief, during proactive efforts, employees are more likely to produce valuable, innovative ideas and boost their creativity.

Koseoglu et al. [7] examined the moderating role of perceived organizational support of creativity. They ignored the influence of how employees’ characteristics and other individual differences respond to creative leadership. Conformity value is defined as employees’ tendency to adapt to social norms and restraint of actions that would violate social expectations [24]. Traditionally, research has indicated that individuals who exhibit high levels of compliance may be less inclined to challenge the status quo [25]. Additionally, some studies have suggested that conformity has an inhibiting effect on creativity [26,27]. However, these findings overlook the crucial role that leaders play in inspiring and motivating employees to engage in proactive behavior and unleash their creative potential. Social learning theory emphasizes that different individuals learn differently in the same context. In the context of creative leadership, where leaders are known for their innovative and change-oriented approaches, employees with different levels of conformity value may respond differently. Employees who endorse conformity value show more obedience, respect, and trust for leaders [24]. When exposed to creative leaders who constantly search for better ways to promote an organization and challenge the status quo, employees high in conformity value may find themselves autonomously imitating these behaviors and displaying more proactive behavior.

The study makes three contributions. First, creativity’s definition emphasizes the inseparability of creativity from practice [4]. Based on this, we examine the mediating role of proactive behavior between creative leadership and employee creativity. Through this examination, we get a new perspective of the underlying mechanisms for leaders to fuel employee creativity in terms of shaping behavior, no longer limited to exploring the mediating role of psychological mechanisms. Second, we examine the moderating effect of conformity value for employee creativity. Based on social learning theory, we confirm that conformity value, which is generally perceived to be detrimental to creativity, can also present proactivity and creativity, especially under the stimulation of creative leaders. The findings not only break the stereotype of conformity, but also allow a comprehensive understanding of when the impact of creative leadership will be more effective. Third, we make several contributions to proactive behavior research. Most of the existing literature has focused on exploring antecedents of proactive behavior [28,29]. Creativity results in new ideas and solutions [30], which also means improving the status quo and focusing on the future. This is consistent with the starting point of proactive behavior. So we firstly apply proactive behavior in the creativity domain, considering it as an antecedent of creativity as well as a mechanism by which leaders enhance employees’ creativity, which deepens the understanding of proactive behavior.

## 2. Theoretical Background and Research Hypothesis

### 2.1. The Impact of Creative Leadership on Proactive Behavior

Change-oriented behaviors performed by employees to enhance organizational performance are referred to as proactive behavior [16]. Research has found that leaders’ behaviors and attitudes influence employees’ proactive behaviors [31,32]. Based on this, it is reasonable to expect that creative leaders, who demonstrate creativity and appreciate updates during the work process, can effectively promote employees’ proactive behavior.

Firstly, the most significant characteristic of creative leadership is that these leaders are accustomed to defining, identifying, and solving problems in an innovative and useful way [19,33]. They often get involved in discussions about new working methods and are open-minded to new ideas from their employees [34]. This means that they appreciate “change” towards the status quo and prefer to find better solutions to improve results [18]. According to social learning theory [17], as the most important role models in the workplace, leaders provide plenty of opportunities for employees to observe, learn, and imitate. When employees observe that their leaders are always seeking better solutions and encouraging new ideas, they are likely to initiate change for work systems and organizations by practicing proactive behavior, which can bring improvement to the current situation [35].

Besides this, the biggest risk for the initiator of proactive behavior is that it is not always appreciated by leaders, who sometimes perceive it as a threat and may be distant to the initiator or even ostracize him/her at work [36]. This can lead to apprehension in practicing proactive behavior. In contrast, for creative leaders, when employees propose new or creative solutions, they do not see them as a threat, much less harshly criticize them, and try to find value in and explore even the less promising ideas [23]. This attitude not only increases employees’ psychological security for causing change but also encourages them to proactively identify problems and seek better ways to solve work challenges. When feeling obvious encouragement and support, employees will use their professional strengths and practice more proactive behaviors.

Lastly, while the starting point of proactive behavior is to bring positive changes and make organizations more competitive, there is a possibility of failure which may bring loss to the organization [37]. When employees think more about the negative impact of proactive behavior, their motivation to practice it is greatly reduced. Creative leadership, however, has a positive attitude toward failure and considers it as an opportunity to learn [38]. Thus, creative leaders are more likely to convey the faith for employees that failures can be overcome, and motivate them to persevere in the face of setbacks [20]. According to social learning theory, employees can learn their leaders’ attitudes towards failure through observation and internalize this positive attitude as their own values, which can relieve them of the pressure to practice proactive behavior and enable them to focus more on the benefits of the proactive behavior rather than the costs of the failure. It is also beneficial for employees to practice proactive behavior. Based on the above, we propose the following hypothesis.

**H1.**  *Creative leadership is positively related to employees’ proactive behavior.*

### 2.2. The Impact of Proactive Behavior on Employees’ Creativity

Creativity describes how individuals develop innovative and valuable ideas, services, or processes in a social system, which places much emphasis on being action-based [39]. Proactive behavior refers to the actions taken by employees on their own initiative that are future-oriented, aiming to improve the working system [22]. What creativity and proactivity have in common is that both focus on innovation at work and efforts towards a warmer and more productive workplace. So we suggest that the process of practicing proactive behavior helps to boost creativity.

Above all, proactive behavior is a type of positive behavior that aims to improve the status quo. After noticing a problem, proactive employees take the initiative to find a solution and apply it rather than leave it be. While practicing proactive behavior, employees work out effective solutions to improve the situation through continuous attempts [40]. During the process, employees also sharpen their creativity, because the creative efforts are directed at innovative and valuable ideas that are not only different from existing ideas, but also contribute to a better situation in a real sense. Obviously, creativity training is realized in the process of proactive behavior.

Besides this, based on the componential model of creativity, the formation of creativity consists of three major components: domain-relevant skills, creativity-relevant skills, and intrinsic motivation [23]. First, Amiabile believed that creativity cannot be developed without the accumulation of domain-relevant skills. When employees are eager to rectify problems in an organization, such as streamlining a process or updating working methods, they need to draw on domain-relevant skills. Proactive employees take advantage of every opportunity to exceed normal job expectations and update their knowledge and skills [39], which certainly will enhance their domain-relevant skills.

As for creative-relevant skill, this can also be acquired through proactive behavior. Proactive employees tend to take self-initiated actions to go beyond normal job requirements [41]. During the process, they are more likely to discover problems and seek feasible solutions. They try to analyze problems from new perspectives, break out of well-used sets, and complete tasks in unusual and non-standard ways [35,42]. In addition, Amiabile [23] pointed out that creative-relevant skills depend on perseverance in the face of frustration; proactive behavior initiators are known as risk-takers who own a higher tolerance of setbacks. When problem-solving strategies are proved unsuccessful, they will move off in a new direction immediately instead of getting disillusioned, which is conducive to the promotion of their creative-relevant skills.

In the end, initiators of proactive behavior are motivated to identify and solve potential problems due to their focus on the task itself, not for external rewards or accolades, which proves their high intrinsic motivation [43]. In addition, research has shown that high intrinsic motivation drives individuals to focus on challenging goals, while low levels trigger individuals to focus on safe and conservative goals [44]. Initiators proactively changing the status quo in an organization rather than passively accepting it [45] confirm that they own a high intrinsic motivation level once again, which is also an important component of creativity.

To sum up, in terms of the outcome of creative efforts, proactive behavior promotes the generation of valuable, innovative ideas and solutions, hence improving employee creativity; in terms of the components of creativity, proactive behavior contributes to the formation of the three components. Therefore, we argue that practicing proactive behavior is beneficial to employee creativity. Based on the above, we propose the following hypothesis.

**H2.**  *Proactive behavior is positively related to employees’ creativity.*

### 2.3. The Mediating Role of Proactive Behavior

To sum up, based on social learning theory, we suggest that employees are more likely to present more proactive behaviors under creative leadership by observing and learning as well as imitating their attitudes and behaviors, which ultimately enhances their creativity level, as creativity places much emphasis on being action-based. By integrating these arguments, we come to the third hypothesis that suggests that the impact of creative leadership on employee creativity will be mediated by proactive behavior [7,10,11,12]. Thus, we hypothesize the following:

**H3.**  *Proactive behavior will mediate the effect of creative leadership on employees’ creativity.*

### 2.4. The Moderating Role of Conformity Value

Employees with a higher conformity value show a higher level of obedience and respect for authorities [24]. They are more likely to obey leaders’ behavioral values. According to social learning theory [17], creative leaders, who are considered the representative role models in a workplace, can influence these employees by demonstrating proactive behavior themselves. When employees find that their leaders do not stick to the status quo and always do things better to improve results [46], their values of deference to authority lead them to behave like leaders, bringing beneficial changes to the work environment and practicing more proactive behavior.

Conformity-oriented employees tend to place a high value on trust and compliance with authority figures [47]. When they perceive that creative leaders endorse and support proactive behavior, they are more likely to trust the leaders’ judgment and have confidence in the effectiveness of such behavior. This trust and confidence acts as a motivational factor that encourages employees with a higher conformity value to step out of their comfort zone and engage in proactive actions.

Employees with a higher conformity value have a stronger inclination to adhere to social expectations and norms as well as prioritize fitting in and being accepted by their social groups [24,27]. When creative leadership conveys a signal of the appreciation of proactive behavior and promotes it as an acceptable and expected norm within the organization, these employees may perceive that engaging in such behavior can help them to enhance their social acceptance. This sense of belonging and acceptance can motivate them to align their behavior with the expectations set by creative leadership.

Conformity-oriented employees appreciate clear guidelines from authority figures [48]. Creative leaders who communicate their expectations regarding proactive behavior provide clear guidelines for employees with a higher conformity value to follow. This clarity reduces ambiguity and uncertainty, making it easier for these employees to understand what proactive actions are expected from them and how they can contribute to the organization’s goals.

In contrast, individuals who value low levels of conformity present a lower level of dependence and submission to leaders [24]. They do not pay special attention to the way leaders behave. It should be noted that employees with a low conformity value can also find leaders’ preference through leaders’ behavior. But they are characterized by a higher focus on self-interest and not being constrained by existing norms and expectations. This may lead them to be less prone to reacting to leaders’ appreciative behaviors. According to social learning theory [17], under the same situation, the higher level an observer gets approval for a model, the better the “learning” effect is presented. Although they all work with creative leaders, compared with employees of a high conformity value, low-conformity-value individuals are less likely to practice proactive behaviors. The above theorizing suggests that conformity value may function as a moderator to accentuate the positive relationship between creative leadership and proactive behavior. Thus we hypothesize the following:

**H4.**  *When conformity value serves as a moderator between creative leadership and proactive behavior, the relationship is stronger for employees with a high conformity value than for those who have a low level of conformity value.*

Combining Hypothesis 3 and Hypothesis 4, we consider that conformity value plays a moderating role in the indirect effect of creative leadership on employee creativity via proactive behavior [7,9,13]. Specially speaking, for employees with a high-level conformity value, the relationship between creative leadership and proactive behavior is more significant, so the indirect influence of creative leadership on employee creativity can also be strengthened. Conversely, for individuals who own a low level of conformity, the influence of creative leadership on proactive behavior will be reduced, which also weakens the indirect effect of creative leadership on employee creativity transmitted by proactive behavior.

**H5.**  *Conformity value can moderate the mediating effect of proactive behavior between creative leadership and employees’ creativity. The effect is stronger for employees with a high conformity value than for those who have a low conformity value.*

**Figure 1:**
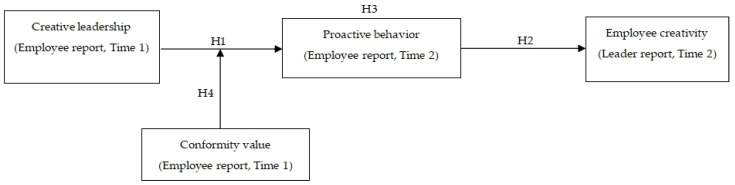
Conceptual Model.

In summary, the conceptual model of this study is shown in Figure 1.

## 3. Methods

### 3.1. Sample Collection and Procedure

Research data were obtained from a large automobile manufacturing enterprise in southern China. We selected their five R&D departments as our target subjects. Their daily duties are related to innovation, such as designing new vehicles, testing trial products, and improving industrial technology and industrial incubation. Employees in these departments are encouraged to be creative because they need to keep up with trends in technology and raise and implement novel designs and proposals to attract consumers.

In order to ensure the process of sample collection and avoid deviation from the common method [49], we have collected survey data matched between employees and their direct leaders for two periods. The process is specified as follows:

1. With the help of the Human Resource Department, we explained the purpose of the study to participants and ensured that the data would only be used for academic research. Before data collection, we obtained the names of the survey targets (employees and their direct leaders) in advance from the HR department and coded them for the use of data matching. In total, 288 employees and 40 leaders voluntarily participated in the survey.
2. We collected data by distributing paper questionnaires on site during the employees’ paid working hours. We also issued envelopes for employees who participated in surveys to be sealed on site in order to practice the principle of confidentiality.
3. At time 1, we invited targeted employees to assess their leaders’ creativity, their own conformity value, and their demographic information (gender, age, and leader–follower dyad tenure). Two weeks later, at time 2, secondary questionnaires were distributed to participants for measuring their proactive behavior. At the same time, we invited department leaders to rate their subordinates’ creativity level.
4. Questionnaires with too many gaps and too-consistent response tendencies were eliminated. For the completed questionnaires of employees, removal criteria are described below: (1) for the first questionnaire, which evaluates creative leadership and conformity value, all questions in the two sections are given the same score, or although score differentiation exists between the two sections, questions in the same section are given the same score; (2) for the second questionnaire, which surveys proactive behavior, all questions are given the same score, revealing that those respondents were not serious about the survey and only ticked an answer casually, so they were therefore removed from our collection. We removed 32 employee questionnaires in total. Among the 40 leaders that we intended to survey, 4 did not fill out the questionnaire because they were on a business trip, and 2 rated each subordinate the same score (1 point), showing that they did not provide a valid evaluation of employees’ creativity, so were removed from our collection. Correspondingly, the completed questionnaires of 26 employees under the aforementioned 6 leaders were also deleted.

In total, we obtained 230 effective leader–employee matched questionnaires from 230 employees (survey response rate = 79.86%) and 34 leaders (survey response rate = 85%). Each leader is matched with 6.76 employees. Table 1 displays the descriptive statistics of the respondents.

### 3.2. Variable Measurement

Since the scales used in the questionnaire were all from English versions, we translated all scales into Chinese according to Harkness et al.’s (2004) [50] translation–back-translation procedure. All the surveys were rated utilizing a 5-point Likert scale ranging from 1 (strongly disagree) to 5 (strongly agree). We provide an overview of all the items in the Appendix A.

Creative leadership. We used the 4-item scale from Farmer et al., (2003) [51] to measure creative leadership [7]. One typical item was “My leader tries new ideas or methods first”. The Cronbach’s alpha was 0.95.

Proactive behavior. We used a 3-item scale from Griffin et al., (2007) [52] to measure proactive behavior. One typical item was “I initiate better ways of doing my core tasks”. The Cronbach’s alpha was 0.92.

Employees’ creativity. We used a 13-item scale from Zhou and George (2001) [53] to measure employees’ creativity. One typical item was “This employee is a good source of creative ideas”. The Cronbach’s alpha was 0.92.

Conformity value. We used a 4-item scale from Miron et al., (2004) [54] to measure conformity value. One typical item was “I think people should know how to obey orders”. The Cronbach’s alpha was 0.89.

Control variables. As for demographic information, employee and leader participants provided information about their gender (1 for female, 2 for male), age, and leader–follower dyad tenure, which are well known for their influence on employee creativity [20,55].

## 4. Data Analysis and Research Results

### 4.1. Common Method Variance Test and Confirmatory Factor Analysis

Before testing specific hypotheses, we conducted confirmatory factor analyses (CFA) with Mplus 8.3 to establish discriminant validity between the research variables. CFA results are presented in Table 2.

As we can see from the results, the hypothesized four-factor model (i.e., creative leadership, proactive behavior, conformity value, and employees’ creativity) is an acceptable fit to the data. Thus, the hypothesized model is more suitable than any other alternative model. The result also confirmed that the construct validity and discriminant validity of the measures used in this study are acceptable. (χ^2^/df = 2.69, CFI = 1.515, TLI = 0.947, RMSEA = 0.047, SRMR = 0.044.)

In addition, although this paper adopts the multi-time point pairing method to collect data to avoid the problem of common method bias, we still consider it necessary to summarize the validity of the test data from the test results. Harman’s single potential factor method was firstly used in this study to test common method bias. Unrotated exploratory factor analysis was conducted together with the topics of creative leadership, proactive behavior, conformity value, and creativity. Results showed four factors with eigenvalues greater than 1, and the first factor had a variance of 33.455%, which is below 40%, showing that common method bias is not a problem in these data. In addition to Harman’s technique, we adopted controlling for unmeasured latent factor methods to test common method bias [56,57]. The results showed that when we added the common method factor to the hypothesized four-factor model, the indicators CFI, TLI, RMSEA, and SRMR did not improve significantly (χ^2^/df = 1.44, RMSEA = 0.044, SRMR = 0.040, CFI = 0.963, TLI = 0.955), further indicating that common method bias is not a problem for the data.

### 4.2. Descriptive Statistics and Correlation Analysis

The means, standard deviations, and correlations of the research variables are shown in Table 3. From these results, we can see that creative leadership is positively linked with both proactive behavior (r = 0.388, *p* < 0.01) and employees’ creativity (r = 0.351, *p* < 0.01). Furthermore, the results also present a positive effect of proactive behavior on employees’ creativity (r = 0.539, *p* < 0.01). Therefore, there is preliminary support for the hypothesis. In addition, conformity value is also positively related to creative leadership (r = 0.371, *p* < 0.01) and proactive behavior (r = 0.330, *p* < 0.01).

### 4.3. Hypothesis Test

#### 4.3.1. Main Effect and Mediating Effect Test

Although our study variables were conceptualized and measured at the individual level, our data had a nested structure because our sample consisted of multiple participants who reported to the same leader. Therefore, we calculated interclass correlation coefficients (ICCs) to check whether our data were conducive for multilevel analysis [58]. The estimated ICC(1) for employees’ creativity was 0.017; this suggested that only 1.7% of the variance in creativity was explained by employees’ group membership. This was below the standard of ICC(1) > 0.05 [59]. So the use of multilevel modeling was unsuitable. Based on this, the study employs the statistical software SPSS 27.0 and the Process 4.1 plug-in to test the effect of creative leadership on employees’ creativity and the mediating effect of proactive behavior. Employees’ and leaders’ gender and age as well as leader–follower dyad tenure are included in the regression model as control variables.

We first used hierarchical multiple regression analysis to test the main effect (Hypothesis 1 and 2) and the mediating effect (Hypothesis 3). Firstly, we tested the effect of creative leadership on employees’ proactive behavior; then we tested the effect of employees’ proactive behavior on their creativity level. In order to confirm the mediating role of proactive behavior, we also tested the role of creative leadership in influencing employees’ creativity levels, and then we tested whether the influence of creative leadership on employees’ creativity weakens or disappears when proactive behavior enters the model.

The results of hierarchical regression analyses are shown in Table 4. Hypothesis 1 proposed that creative leadership was positively related to employees’ proactive behavior. Model 2 showed that creative leadership was significantly associated with employees’ proactive behavior (β = 0.235, *p* < 0.01), which supported Hypothesis 1. Hypothesis 2 proposed that employees’ proactive behavior was positively related to their creativity level. In Table 4, Model 7 demonstrated that proactive behavior was positively linked to employees’ creativity (β = 0.555, *p* < 0.001), so Hypothesis 2 was also substantiated. Hypothesis 3 proposed that proactive behavior played a mediating role between the relationship of creative leadership and employees’ creativity. Table 4 showed the following: (1) creative leadership was positively related to proactive behavior; (2) proactive behavior was positively related to employee creativity; (3) creative leadership was positively related to employees’ creativity (β = 0.262, *p* < 0.001, Model 6); (4) when proactive behavior began, the positive influence of creative leadership on creativity decreased from 0.262 (*p* < 0.001) to 0.147 (*p* < 0.01) (Model 8), supporting the partial mediation of proactive behavior [60].

In addition, we used mediated regression analyses with bias-corrected bootstrapping for indirect effects [61]. The results in Table 5 show that the indirect effect of creative leadership on employee creativity via proactive behavior was 0.115 and the 95% bias-corrected confidence interval around the bootstrapped indirect effect did not contain zero (CI = [0.051, 0.198]). So Hypothesis 3 was supported.

#### 4.3.2. Moderating Effect Test

For this section, we first employed multiple regression analysis to explore whether conformity value moderates the influence of creative leadership on proactive behavior. We added the interaction term of creative leadership and an independent conformity value of centralization processing into the model. The existence of the moderating effect can be confirmed if the regression coefficient of the interaction term is significant. As we can see in Table 4, Model 4 showed that the interaction between creative leadership and conformity value was significantly towards proactive behavior (β = 0.161, *p* < 0.05).

**Figure 2:**
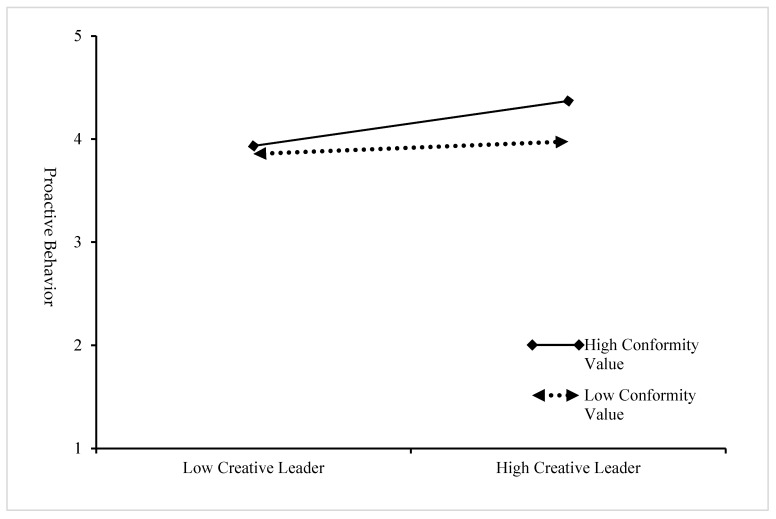
The moderating effect of conformity value on the relationship between creative leader and employee proactive behavior.

In addition, to better explain the moderating effects of conformity value, the sample was divided into high- and low-conformity-value groups (mean conformity value ± one standard deviation), and the moderating effect of conformity value was plotted using simple slope analysis. As shown in Figure 2, for subjects with a low conformity value, the positive effect of creative leadership on proactive behavior was not significant (β = 0.058, n.s.), whereas for subjects with a high conformity value, this relationship was positively significant (β = 0.317, *p* < 0.001), indicating that conformity value enhanced the positive effect of creative leadership on proactive behavior and that this positive effect was only for employees with higher levels of conformity value.

#### 4.3.3. The Test of Moderated Mediation Effect

To test the moderated mediation relationship suggested in Hypothesis 5, we followed the approach outlined by Hayes [61]. As recommended by Hayes [61], we examined the statistical significance of the conditional indirect effect at one SD below and one SD above the mean for conformity value. Table 6 shows that the indirect effect of creative leadership on employees’ creativity was significant at one SD above the mean for conformity value (indirect effect = 0.135, SE = 0.042, CI [0.062, 0.225]) but non-significant when it was one SD below the mean for conformity value (indirect effect = 0.037, SE = 0.036, CI [−0.028, 0.113]). Besides this, the index of moderated mediation was also significant (index = 0.079, SE = 0.038, CI [0.010, 0.159]). So Hypothesis 5 was supported.

## 5. Discussion and Conclusions

In this study, we present a moderated mediation model to explore how and when creative leaders stimulate employee creativity. Our findings indicate that creative leaders are better at promoting employees’ proactive behavior, which in turn enhances employee creativity. In addition, employees with a high conformity value are more likely to be influenced by creative leaders to present more proactive behaviors. Furthermore, the indirect effect of creative leaders on employee creativity via proactive behavior can be strengthened in the presence of employees’ high conformity values. These findings yield meaningful theoretical as well as practical implications.

### 5.1. Theoretical Implications

The study contributes several theoretical implications. First, obeying the well-known truth “action speaks louder”, we consider proactive behavior as the transmission mechanism through which creative leadership enhances employee creativity. Existing research has capitalized on a psychological perspective to clarify the underlying influence of leaders on employee creativity [55,62]. We provide a new theoretical perspective of leaders in shaping employees’ behavior for fueling their creativity beyond the existing underlying psychological perspective. Our findings suggest that observing leaders’ creative actions can trigger employees’ learning and imitating processes to demonstrate more proactive behavior and ultimately promote creativity. On this point, we also make some contributions to social learning theory. Generally speaking, the application of social learning theory mainly focuses on how leaders shape employees’ behaviors, taking employees’ behaviors as the final result of social learning [63]. In our research, we propose that employees practice proactive behavior after observing, learning, and imitating role models (creative leaders), which will eventually facilitate their creativity. Proactive behaviors are only the explanatory mechanism of the creative leadership–creativity relationship, not the final learning outcomes; the research ultimately comes down to employee creativity, expanding the application of social learning theory.

Second, the research not only extends the leadership and creativity literature but also helps us to gain a deeper understanding of the vital role that social learning processes plays in leader–employee interactions. Based on the theory, we demonstrate the boundary condition of the effect of creative leaders on employee creativity–conformity value. We found that conformity value can serve as a contextual factor in strengthening creative leaders’ positive impact on creativity. When employees own a high-level conformity value, creative leaders are more likely to enhance their creativity through promoting proactive behavior. This finding confirms that conformity value and creativity are not always paradoxical, but rather can coexist harmoniously, especially under the context of creative leadership. According to the research results, we provide a novel insight about the role of conformity played in creativity, which is always seen as negative [26], confirming that social learning processes can change the interplay of leadership, individual personality, and individual behavior, and sometimes brings unexpected results.

Third, we expand proactive behavior research by firstly applying it in the creativity domain. In the little extant research exploring the outcomes of proactive behavior, no attention was paid to the effect of proactive behavior on creativity [37]. Practicing proactive behavior requires employees to take the initiative to bring positive change to current situations [15]. The implementation process is conducive to the generation of new and valuable solutions, in which creativity can also be boosted. So we consider creativity as a positive result produced by proactive behavior and tested its mediated role between creative leaders and creativity, providing new research directions for proactive behavior.

### 5.2. Practical Significance

Creativity is seen as a “potent competitive weapon” for organizations. Considering the importance of creative leadership in facilitating employees’ creativity, we call on organizations to invest more focus on creative leadership. Often when organizations choose employees for higher roles, they generally prefer those who do not take risks to creative or risk-taking employees [19]. However, our research suggests that organizations should promote creative leaders because these kinds of leaders could effectively stimulate employees’ creativity through proactive behavior. So the establishment of an effective promotion system for them is necessary within organizations. Specifically, how creative these employees have been in the past may need to be considered before they are promoted to be leaders, since this may play an important role in influencing the level of all of their subordinates’ creativity. Besides this, in order to improve creative leadership, organizations could also encourage training programs for leaders in order to improve their creativity, such as how to recognize opportunities and adapt to changes in their work environment. As we all know, leaders could be important sources of employee behavioral modeling; one way that creative leadership may be able to influence employees’ creativity is by actually being creative at work themselves, so we encourage creative leaders to present their creativity as much as possible in the workplace. For instance, they could influence employees’ creativity by sharing their expertise, assigning appropriate tasks, providing resources and rewards, connecting employees to external contacts, giving feedback, and stimulating their employees [64]. This could facilitate employees’ proactivity and ultimately enhance their creativity. Also, research has found that having creativity goals improves creative performance [65]. Given this, organizations could encourage leaders to set creativity goals for themselves, since this could potentially be associated with higher levels of employee creativity. Additionally, organizations should pay attention to designing a work environment that their employees would find to be supportive of creativity; this type of organizational support for employees may allow them to fully act on their propensity to be creative, potentially taking risks, experimenting, and trying to generate more creative ideas and products.

Besides this, the uncovered moderating role of conformity value between creative leadership and employee creativity suggests that organizations should find the optimum balance of conformity-oriented employees towards creativity and arrange them to work under creative leaders. The research confirms that it is a win–win situation for both leaders and employees.

### 5.3. Limitations and Prospects

Despite its strengths, the study also has some limitations that call for more future research. First, since the effectiveness of leaders depends on the context [66], we should be cautious about the generalizability of the findings. This study was conducted in a creativity-oriented department (i.e., R&D) where creative leaders are more appropriate and beneficial. In addition, all the data were collected in China, a country with relatively centralized power. When the role model is a leader, employees are more willing to imitate them. In order to determine whether the findings are applicable in other contexts, we call for future research to take other cultures and industries into account.

Second, we used cross-sectional data in this research so that we cannot infer causality [49]. Another explanation for our findings is that creative leaders prefer to select employees who are also creative. In the future, longitudinal research could be conducted in order to explore the extent to which creative leaders select creative employees vs. the extent to which creative leaders facilitate such employees.

Third, the findings demonstrate that proactive behavior only partially mediates the effect of creative leaders on employee creativity, confirming the existence of additional potential mediators. For example, from a behavioral perspective, we suggest that creative leaders may engage in contingent reward behavior [67] to motivate employees’ creative performance. We recommend that future research examines this and other potential mediating mechanisms to comprehensively explain the influence of creative leaders on employee creativity.

Finally, our model focuses on the impact of leaders’ type, employees’ behaviors, and characteristics on employee creativity. We do not take work or organizational factors into the consideration. However, these two factors have been proven to be related to employee creativity [59]. Future studies could examine more deeply the ways these factors interact together to influence the creative leader–employee creativity relationship.

### 5.4. Conclusions

Extending research on leadership and creativity, the present study highlights the salient role of creative leadership in facilitating employee creativity. Specially, drawing from social learning theory on creativity research, this research examines the mechanism whereby creative leadership contributes to employees’ creativity and a boundary condition under which this effect of creative leadership is more pronounced. These findings reveal the process underlying employee creativity from a behavioral perspective. That is, creative leadership is more likely to spur employees’ proactive behavior by influencing employees’ social learning processes, and, as a result, improve their creative performance, with this indirect effect being stronger in the presence of employees’ higher conformity values. Our hope is that this study will spark continued interest in exploring the influence of leaders on important follower outcomes, including but not limited to employee creativity.

## Figures and Tables

**Table 1:** Sample demographic characteristics distribution.

Characteristic	Form	N	%
Gender of employee	Male	97	42.2%
	Female	133	57.8%
Age of employee	21–30	131	57%
	31–40	72	31.3%
	41–50	27	11.7%
Gender of leader	Male	11	32.4%
	Female	23	67.6%
Age of leader	31–40	7	20.6%
	41–50	18	52.9%
	51–50	9	26.5%
Leader–follower dyad tenure	2–5	72	31.3%
	6–9	118	51.3%
	10 years above	40	17.4%

**Table 2:** Results of confirmatory factor analysis.

Variables	χ^2^	df	χ^2^/df	TLI	CFI	RMSEA	SRMR
Hypothesized four-factor model	444.027	293	1.515	0.947	0.952	0.047	0.044
Three-factor model (CL and CV combined)	847.269	296	2.862	0.807	0.824	0.090	0.091
Three-factor model (PB and CV combined)	893.246	296	3.018	0.791	0.809	0.094	0.102
Three-factor model (CL and PB combined)	1050.217	296	3.548	0.736	0.759	0.105	0.111
Two-factor loading (CL, PB and CV combined)	1440.554	298	4.834	0.602	0.635	0.129	0.121
One-factor loading	1783.163	299	5.964	0.485	0.526	0.147	0.135
Hypothesized four-factor model + CMV	384.607	267	1.440	0.963	0.955	0.044	0.040

Note(s): CL = creative leadership; PB = proactive behavior; CV = conformity value. Abbreviation(s): df, degrees of freedom; TLI, Tucker–Lewis index; CFI, comparative fit index; RMSEA, root-mean-square error of approximation; SRMR, standardized root-mean-square residual.

**Table 3:** Descriptive statistics and correlation coefficients of each variable.

Variable	M	SD	1	2	3	4	5	6	7	8
1. Creative leadership	3.424	0.795	**(0.882)**							
2. Proactive behavior	4.047	0.592	0.338 **	**(0.901)**						
3. Creativity	3.861	0.608	0.351 **	0.539 **	**(0.896)**					
4. Conformity value	3.407	0.617	0.371 **	0.330 **	0.366 **	**(0.862)**				
5. Employee gender	1.578	0.495	−0.147 *	−0.133 *	−0.054	−0.214 **	-			
6. Employee age	31.465	7.435	0.069	0.158 *	0.142 *	0.041	−0.192 **	-		
7. Dyad tenure	4.613	3.544	0.107	0.190 **	0.148 *	0.119	−0.106	0.791 **	-	
8. Leader gender	1.343	0.476	0.002	0.066	0.023	−0.102	0.284 **	−0.263 **	−0.206 **	-
9. Leader age	40.778	6.23	0.096	−0.013	0.066	0.086	0.094	0.418 **	0.453 **	−0.023

Notes: N = 230. Gender: 1 = male, 2 = female. * *p* < 0.05. ** *p* < 0.01. Reliability coefficients are reported in bold on the diagonal.

**Table 4:** Results of hierarchical regression analyses.

	Proactive Behavior				Creativity			
Variables	M1	M2	M3	M4	M5	M6	M7	M8
Employee Gender	−0.161 *	−0.095	−0.044	−0.051	−0.062	0.012	0.027	0.058
Employee Age	0.003	0.006	0.010	0.011	0.006	0.009	0.004	0.006
Dyad Tenure	0.038 *	0.031	0.024	0.020	0.018	0.010	−0.004	−0.005
Leader Gender	0.199 *	0.177 *	0.198 *	0.209 **	0.099	0.074	−0.012	−0.013
Leader Age	−0.011	−0.014 *	−0.016 *	−0.016 *	0.000	−0.004	0.006	0.003
Creative Leadership		0.235 **	0.174 ***	0.175 ***		0.262 ***		0.147 **
Proactive Behavior							0.555 ***	0.490 ***
Conformity Value			0.234 ***	0.191**				
Creative Leadership* Conformity Value				0.161*				
R^2^	0.080	0.176	0.224	0.240	0.030	0.142	0.298	0.329
ΔR^2^	0.080	0.095 ***	0.048 ***	0.017 *	0.030	0.112 ***	0.268 ***	0.187 ***
F	3.920 **	7.915 ***	9.149 ***	8.744 ***	1.375	6.148 ***	15.758 ***	15.577 ***

Notes: N = 230 * *p* < 0.05. ** *p* < 0.01.*** *p* < 0.001.

**Table 5:** Results of the bootstrapping analysis for mediating effect.

Mediation				
Mediating Effect				
Creative Leadership→Proactive Behavior→Creativity	Indirect Effect	BootSE	Boot LLCI	Boot ULCI
	0.115	0.038	0.051	0.198

Abbreviations: LLCL, lower limit of confidence interval; ULCL, upper limit of confidence interval.

**Table 6:** Results of bootstrapping analysis for moderated mediation analysis.

Moderated Mediation					
95% Bias-Corrected Confidence Intervals					
Dependent Variable	Level of Moderator	Effect	BootSE	Boot LLCI	Boot ULCI
Creativity	Low (−1 SD)	0.037	0.036	−0.028	0.113
	Mean	0.086	0.032	0.032	0.155
	High (+1 SD)	0.135	0.042	0.062	0.225
	Index of Moderated Mediation	0.079	0.038	0.010	0.159

Abbreviations: LLCL, lower limit of confidence interval; ULCL, upper limit of confidence interval; SD, standard deviation.

## Data Availability

The data presented in this study are available on request from the corresponding author.

## Appendix A

**Survey Items**
**Creative Leader** **ship**

1. My leader tries new ideas or methods first.
2. My leader seeks new ideas and ways to solve problems.
3. My leader generates ground-breaking ideas related to the field.
4. My leader serves as a good role model for creativity.

**Proactive Behavior**

1. I initiate better ways of doing my core tasks.
2. I come up with ideas to improve the way in which my core tasks are done.
3. I make changes to the way my core works are done.

**Conformity Value**

1. I try not to oppose team members.
2. I adapt myself to the system.
3. I adhere to accepted rules in my area of work.
4. I avoid cutting corners.

**Employee Creativity**

1. This employee suggests new ways to achieve goals or objectives.
2. This employee comes up with new and practical ideas to improve performance.
3. This employee searches out new technologies, processes, techniques or product ideas.
4. This employee suggests new ways to increase quality.
5. This employee is a good source of creative ideas.
6. This employee is not afraid to take risks.
7. This employee promotes and champions ideas to others.
8. This employee exhibits creativity on the job when given the opportunity to.
9. This employees develops adequate plans and schedules for the implementation of new ideas.
10. This employee often has new and innovative ideas.
11. This employee comes up with creative solutions to problems.
12. This employee often has a fresh approach to problems.
13. This employees suggests new ways of performing work tasks.
